# Psychometric properties and factor structure of the Korean version of the screen for child anxiety related emotional disorders (SCARED)

**DOI:** 10.1186/s12888-020-02505-3

**Published:** 2020-02-28

**Authors:** Jiyoon Shin, Kyoung Min Kim, Kyung Hwa Lee, Soon-Beom Hong, Jung Lee, Chi-Hyun Choi, Ji Youn Han, Seong Hae Kim, Da Eun Suh, Soo-Churl Cho, Jae-Won Kim

**Affiliations:** 1 Division of Child and Adolescent Psychiatry, Department of Psychiatry, Seoul National University College of Medicine, 103 Daehak-Ro, Chongno-Gu, Seoul, 03080 Republic of Korea; 2 Department of Psychiatry, Dankook University College of Medicine, Cheonan, 31116 Republic of Korea; 3 Integrative Care Hub, Seoul National University Children’s Hospital, Seoul, 03080 Republic of Korea; 4 Department of Psychiatry, Seoul National University Boramae Medical Center, Seoul, 07061 Republic of Korea; 5 Department of Psychiatry, Korea Armed Forces Capital Hospital, Bundang, Republic of Korea

**Keywords:** SCARED, Validity, Reliability, Factor structures, Anxiety disorder

## Abstract

**Background:**

The aim of this study was to examine the psychometric properties of the Korean version of Screen for Child Anxiety Related Emotional Disorders (SCARED) on a sample of Korean youths and to examine the cross-cultural differences in adolescents’ anxiety.

**Methods:**

Our study included 147 adolescents (ages 12–17, 92 girls), 93 with major depressive disorder and 54 as controls. Participants were evaluated using the Kiddie-Schedule for Affective Disorders and Schizophrenia for School-Age Children-Present and Lifetime Version (K-SADS-PL), SCARED, Child Behavior Checklist (CBCL), Disruptive Behavioral Disorder Scale (DBD) and Attention Deficit Hyperactivity Disorder Rating Scale (ADHD-RS). Pearson’s *r* and Cronbach’s α values of the SCARED were calculated, and exploratory factor analysis was conducted.

**Results:**

The Korean SCARED scores were correlated with the total anxiety scores of K-SADS-PL (*r* = 0.74) and the CBCL anxious/depressed subscale scores (*r* = 0.35). Results showed a five-factor structure with good internal consistency, in which some items were loaded on different factors compared to previous studies.

**Conclusions:**

The Korean SCARED demonstrated promising psychometric properties, and could be a valid scale for screening anxiety symptoms in primary care. The fact that different items comprised the factors may reflect the cultural difference between United States and Korea in experiencing anxiety.

## Background

Anxiety disorders are the most prevalent psychiatric disorders in children and adolescents worldwide. In the United States, the point prevalence rate of adolescents’ anxiety disorders based on DSM-IV diagnosis was 15% and the 12-month prevalence rate was as high as 25% [1]. In Korean children and adolescents aged 6 to 18, the one-year prevalence rate of anxiety disorder was 11.7% [2]. Another study of Korean children aged 6 to 12 showed that the past-year prevalence of anxiety disorders was 10.3% [3]. Despite these high prevalence rates, anxiety disorders are often underdiagnosed and remain untreated for the following reasons: first, they are not as evident as behavioral disorders [4]; second, they are frequently comorbid with other psychiatric disorders that gain more clinical attention, such as depressive disorders and substance abuse [5, 6].

In South Korea, there is a cultural emphasis on academic achievement [7], which creates an excessive academic burden, an overly competitive atmosphere, and university entrance-oriented education [8, 9]. As a result, adolescents’ anxiety and depression tend to increase as the grade level increases in schools [8–10]. According to a report by the Korean National Youth Policy Institute, 20.9% of Korean youth have thought about committing suicide in one year mainly due to academic stress and anxiety [11]. Anxiety is known to be an important predictive factor for suicidality [10]. Thus, early detection and intervention of Korean youths’ anxiety disorders are important, considering the severity and consequences of these disorders.

Both the Korean versions of the State-Trait Anxiety Inventory (STAIC) and the Revised Children’s Manifest Anxiety Scale (RCMAS) measure youth anxiety. The STAIC and RCMAS are used to evaluate anxiety in various aspects. The STAIC includes both the state and trait scales. While the state scale measures the current feeling of anxiety, the trait scale measures a more stable and pervasive tendency to experience anxiety. The three subscales of RCMAS assess worry/oversensitivity, fear/concentration, and physiological anxiety. Although the STAIC and RCMAS measure general anxiety symptoms, both have limitations. First, they do not provide information regarding specific anxiety disorders. Second, they have some limitations regarding discriminant validity such that they did not distinguish definitely anxiety disorders from behavioral disorders such as ADHD [12].

The Screen for Child Anxiety Related Emotional Disorders (SCARED) is a screening questionnaire for anxiety disorders in youth. It is a rating scale completed by children or parents. Although it has been used in some clinical studies conducted in Korea [13, 14], the psychometric properties of the Korean version are yet to be determined. The scale has several advantages compared to other anxiety scales mentioned above. First, the SCARED can assess specific anxiety disorders in addition to assessing general anxiety symptoms. Second, it can be applied to a wider age range of youth, from 6 to 19 years [15, 16], compared to the STAIC, which is indicated for youth ages 9–12. Third, the English SCARED shows good psychometric properties, specifically in terms of discriminant validity, and can differentiate children with an anxiety disorder from depression or disruptive disorder-only groups [17]. Internal consistency and factor structure were also shown to be robust when applied to different cultures and races, including European countries, South Africa, China, non-Hispanic White and African Americans, and Arabia [18, 19]. In addition, the SCARED is a self-report measure, which is more practical compared to diagnostic clinical interviews [20]. Given such advantages of the SCARED, it is meaningful to examine the psychometric properties of the Korean version of the SCARED to assist better diagnosis and treatment of anxiety disorders in Korean youth.

Due to an historical background of Confucianism, there exists a strong sense of community in South Korea [21, 22]. Children are often brought up to be considerate of others and to be attentive to how they are viewed by others, a character that is called ‘Nun-chi’ in Korean [22, 23]. In social atmospheres as seen in Korea and Japan, there is a culture-specific social fear regarding concern at offending others, called ‘Taein Kongpo’ in Korean, or ‘Taijin Kyofusho’ in Japanese [24, 25]. In a study of Korean middle and high school students, 13% of middle school students and 14.8% of high school students thought that they would cause harm to others because of their social anxiety [23]. Children and adolescents’ excessive social anxiety cause difficulties in peer relationships and academic ability, school refusal, abandonment of school activities, and deterioration of their psychosocial functions [26, 27]. Due to the cultural specificity of Korea, it is possible that the factor structure of the Korean version of the SCARED might differ from that of the English SCARED.

In this study, we aimed to establish the reliability and validity of the Korean SCARED and investigated the factor structure of the scale. We hypothesized that the Korean SCARED would be a valid and reliable scale to assess anxiety symptoms in Korean youth. In addition, we hypothesized that the cross-cultural differences in experiencing social anxiety would be reflected in the factor structure of the Korean SCARED.

## Methods

### Participants

Our study included 95 depressed participants between the ages of 12–17 years. They were part of a study that investigated the biomarkers of the antidepressant response and suicidal events in depressed youth (NRF-2015R1A2A2A01004501). Major depressive disorder (MDD) diagnoses were made using the Kiddie-Schedule for Affective Disorders and Schizophrenia for School-Age Children-Present and Lifetime Version (K-SADS-PL) [7, 28, 29], which is based on Diagnostic and Statistical Manual of Mental Disorders-5th Edition (DSM-5) [30] criteria. The participants were recruited from the child and adolescent psychiatric outpatient clinic at the Seoul National University Hospital between August 2015 and June 2018.

Participants were included in the study if they were diagnosed with MDD, had symptoms lasting for at least 4 weeks or more without psychotic features, scored 40 or higher on the Children’s Depression Rating Scale-Revised (CDRS-R) [31], and scored 4 or higher points on the Clinical Global Impression-Severity scale [32]. Exclusion criteria were intelligence quotient (IQ) under 70, a history of chronic medical and neurological diseases, diagnosis of psychotic disorders such as schizophrenia, diagnosis of bipolar disorders and developmental disorders such as autism, substance abuse within the past 6 months, a diagnosis of bipolar I disorder among immediate relatives, and taking any psychiatric medication. Totally, 57 healthy controls were recruited via school flyers. They visited our clinic and were initially screened using the K-SADS-PL.

Among the initially recruited 152 subjects, 2 depressed participants and 3 healthy controls withdrew their participation consent and therefore were excluded from the study. Thus, a total of 147 subjects (92 of which were girls) were included in the analyses; these were divided into depression and anxiety comorbid (*n* = 44), depression only (*n* = 49) and control (*n* = 54) groups based on the psychiatric diagnoses from the K-SADS-PL.

The study was approved by the Institutional Review Board for Human Subjects of Seoul National University Hospital. All the parents and youth were provided with detailed information about the study. Written informed consent was obtained from all participants before entering the study.

### Assessments

At baseline, all participants were assessed using the K-SADS-PL, SCARED, Child Behavior Checklist (CBCL), Disruptive Behavioral Disorder Scale (DBD) and ADHD Rating Scale (ADHD-RS).

The SCARED is a 41-item self-report that screens for anxiety disorders and can be completed by child and parent. In our study, the SCARED child version was used. In a validation study of the Korean SCARED, the researcher team translated the original SCARED items into Korean, back-translated them to English by a bilingual researcher, and re-checked the back-translated version. Finally, the scale was confirmed by a researcher with Ph.D. in clinical psychology [33].

During assessment, a psychiatrist (J.-W.K) who was blind to the SCARED scores administered the K-SADS-PL to the participants and their parents. To investigate the correlation between the SCARED and K-SADS-PL anxiety symptoms, we calculated a total score by adding up the following subscale scores as per screen section: panic disorder (1 item), separation anxiety disorder (5 items), social phobia (2 items) and generalized anxiety disorder (4 items).

Parents completed the CBCL, DBD, and ADHD-RS. The CBCL is a 113-item scale measuring six DSM oriented scales- affective, anxiety, ADHD, conduct, oppositional defiant disorder (ODD), and somatic problems [34]. In our study, we used the anxious/depressed subscale, internalizing and externalizing scales of CBCL. The DBD is a 41-item scale used to measure ADHD (18 items), ODD (8 items) and conduct disorder (CD) (15 items) symptoms [35, 36]. In our study, we used the CD subscale to assess behavioral symptoms only. The ODD subscale also shows behavioral symptoms, however, they might not be prominent to the parents if the behavioral problems are only confined to the school or outside the home. Thus, we selected CD subscale, which would be more evident to parents. Each item is rated on a 4-point Likert scale. The ADHD-RS is an 18-item self-report used to screen for ADHD [37]. The scale measures two features of ADHD: inattention and hyperactivity-impulsivity. Each item is rated on a 4-point Likert scale. The ADHD-RS is known to specifically evaluate ADHD symptoms, which can be used to screen, diagnose, and evaluate the effect of treatment for ADHD [37]. For this reason, we chose the ADHD-RS over the ADHD subscales of the CBCL and the DBD.

### Statistical analyses

Demographic variables were analyzed using ANOVA or Chi-square tests. To examine the criterion and discriminant validity of the SCARED, Pearson’s correlation coefficients were measured. ANOVA was used to compare the clinical variables among anxiety + depression (A + D), depression only (D only) and control groups. A Tukey’s honest significance test or Dunnett T3, depending on whether the equality of variances was met or not, was done as a post hoc analysis, to examine the differences in the Korean SCARED total and subscale scores among A + D, D only and control groups.

Our sample size satisfied the minimum required sample size for a factor analysis [38], which allowed us to conduct factor analysis to investigate the factor structure of the Korean SCARED. Since the data was not normally distributed [39], principal axis factoring was used to extract factors. Scree test was used to decide the number of factors. Lastly, promax rotation, a method used for analyzing correlated factors [39], was performed to determine the factor structure. All primary data were analyzed with SPSS version 22.0.

Finally, a sensitivity analysis was conducted to assess the robustness and reproducibility of the factor structure we elicited, using an independent dataset. From the ‘Validation of depression-related scales in child and adolescent psychiatric outpatients’ study (IRB No. 1908–088-1055), the sample consisted of 464 participants (278 of whom were girls) aged 7 to 19, recruited from the child and adolescent psychiatric outpatient clinic of Seoul National University Hospital between August 2015 and July 2019. Inclusion criteria were to have completed a semi-structured interview and the same questionnaires as mentioned above in the Assessment section for the intake assessment. Demographic and clinical data were collected in the same manner. Among the sample, subjects included in the primary factor analyses were excluded. After applying the inclusion and exclusion criteria, the sample consisted of 431 participants (257 of whom were girls) aged 7 to 19. Among them, 262 were diagnosed with MDD, 49 with bipolar disorder, 69 with anxiety disorder, 11 with ADHD, 5 with conduct disorder, 2 with tic disorder, 3 with autism, and 29 with eating disorder. One participant had no psychiatric disorder. Confirmatory factor analysis (CFA) using the maximum-likelihood method was performed on the independent dataset. CFA was conducted with R version 1.2.5019, Lavaan package and internal consistencies were calculated with SPSS version 22.0.

## Results

### Demographics and clinical characteristics

No significant difference was found in age, gender and IQ between the A + D, D only, and control groups. However, there were significant differences between three groups in scores on the Korean SCARED, CBCL internalizing scales and the K-SADS-PL anxiety subscale. Post hoc analyses revealed that CBCL anxious/depressed, CBCL externalizing scales, ADHD and CD scores differed significantly between the D only and control groups, but not between the A + D and D only groups (Table 1).

**Table 1:** Demographic and clinical characteristics of primary dataset

	§ Anxiety + Depression (*n* = 44)	§§ Depression only (*n* = 49)	Control (*n* = 54)	statistics	p-value
Gender, n (%)				χ2 = 1.88	0.39
Male	14 (31.8)	17 (34.7)	24 (44.4)		
Female	30 (68.2)	32 (65.3)	30 (55.6)		
Age, mean (SD)	14.8 (1.7)	14.7 (1.6)	14.4 (1.6)	*F* = 0.80	0.45
IQ, mean (SD)	104.6 (14.0)	106.1 (14.2)	108.5 (11.6)	*F* = 1.09	0.34
Anxiety diagnosis					
Social phobia	15 (34.1)				
Agoraphobia	3 (6.8)				
GAD	28 (63.6)				
Panic	5 (11.4)				
Separation anxiety	3 (6.8)				
PTSD	4 (9.1)				
Specific phobia	1 (2.3)				
†K-SADS-PL_anxiety total	21.0 (4.0)	16.3 (2.4)	12.7 (1.6)	*F* = 105.47	< 0.001
†SCARED	43.6 (15.1)	32.4 (14.9)	10.6 (9.7)	*F* = 79.17	< 0.001
CBCL					
†CBCL_anx/dep	58.8 (16.5)	56.2 (13.3)	43.9 (10.6)	*F* = 17.62	< 0.001
†CBCL_ext	50.1 (11.8)	50.3 (12.7)	41.8 (9.6)	*F* = 9.40	< 0.001
†CBCL_int	67.4 (8.7)	62.0 (9.8)	45.0 (10.5)	*F* = 72.27	< 0.001
†ADHD	8.9 (8.4)	12.1 (11.4)	3.7 (5.2)	*F* = 12.40	< 0.001
†CD	1.8 (3.0)	2.5 (4.0)	0.4 (0.9)	*F* = 7.01	0.001

§ Anxiety + Depression: participants with co-morbid anxiety disorders and MDD

§§ Depression only: participants with MDD only

*GAD* generalized anxiety disorder; *PTSD* post traumatic stress disorder

*SCARED* The Screen for Child Anxiety Related Emotional Disorders

*K-SADS-PL* Kiddie-Schedule for Affective Disorders and Schizophrenia-Present and Lifetime version

*CBCL* Child Behavior Checklist

*CBCL_anx/dep* CBCL anxious/depressed subscale

*CBCL_ext* CBCL externalizing scale

*CBCL_int* CBCL internalizing scale

*ADHD* Attention Deficit Hyperactivity Disorder Rating Scale (ADHD-RS)

*CD* conduct disorder assessed by Disruptive Behavioral Disorder Scale (DBD)

† A + D > D only > Control group

### Criterion validity

As shown in Table 2, the Korean SCARED showed significant correlation with the K-SADS-PL anxiety subscale total scores (r = 0.74, *p* < 0.001). We calculated a total score of the K-SADS-PL anxiety subscales by summing up panic disorder (PD), Separation Anxiety Disorder (SAD), social phobia (SP), and generalized anxiety disorder (GAD) subscale scores. The Korean SCARED scores were correlated with all K-SADS-PL anxiety subscales (*r*s > 0.214, *p*-values ≤0.009), of which the GAD subscale (*r* = 0.75, *p* < 0.001) showed the strongest correlation.

**Table 2:** Correlations among SCARED, CBCL_anx/dep, CBCL_int, CBCL_ext, CD and ADHD

	SCARED	K-SADS-PL_anxiety	CBCL_anx/dep	CBCL_int	CBCL_ext	CD	ADHD
SCARED	1						
K-SADS-PL_anxiety	.74^**^	1					
CBCL_anx/dep	.35^**^	.37^**^	1				
CBCL_int	.57^**^	.60^**^	.78^**^	1			
CBCL_ext	.12	.21^*^	.68^**^	.59^**^	1		
CD	.10	.12	.25^**^	.32^**^	.48^**^	1	
ADHD	.15	.14	.46^**^	.49^**^	.54^**^	.55^**^	1

** *p* < 0.01, * *p* < 0.05

SCARED: The Screen for Child Anxiety Related Emotional Disorders

K-SADS-PL: Kiddie-Schedule for Affective Disorders and Schizophrenia-Present and Lifetime version

*CBCL* Child Behavior Checklist

*CBCL_anx/dep* CBCL anxious/depressed subscale

*CBCL_ext* CBCL externalizing scale

*CBCL_int* CBCL internalizing scale

*ADHD* Attention Deficit Hyperactivity Disorder Rating Scale (ADHD-RS)

*CD* conduct disorder assessed by Disruptive Behavioral Disorder Scale (DBD)

The Korean SCARED and CBCL anxious/depressed subscale also showed significant correlation, although it was relatively low (*r* = 0.35, *p* < 0.001).

### Discriminant validity

The correlation between the Korean SCARED and the externalizing problem of CBCL was not significant (*r* = 0.13, *p* = 0.14). On the contrary, the Korean SCARED and the internalizing problem of CBCL showed moderate correlation (*r* = 0.57, *p* < 0.001). The Korean SCARED scores did not correlate with CD of the DBD (*r* = 0.29, *p* = 0.25), as well as with the ADHD-RS (*r* = 0.15, *p* = 0.07) (Table 2).

### Internal consistency

As shown in Table 3, internal consistencies of five factors (for details, see 5. Factor structure) ranged from 0.89 to 0.94. Overall internal consistency was good (Cronbach’s α =0.96).

**Table 3:** Factor loadings and factor internal consistency (*N* = 147)

Factor	Item		Factor loading	Cronbach’s α
Generalized anxiety				0.91
	5	I worry about other people liking me	1.01	0.90
	21	I worry about things working out for me	0.83	0.90
	33	I worry about what is going to happen in the future	0.80	0.90
	14	I worry about being as good as other kids	0.78	0.90
	23	I am a worrier	0.78	0.90
	28	People tell me that I worry too much	0.74	0.90
	35	I worry about how well I do things	0.74	0.90
	37	I worry about things that have already happened	0.73	0.90
	20	I have nightmares about something bad happening to me	0.61	0.91
	9	People tell me I look nervous	0.55	0.91
	7	I am nervous	0.49	0.91
	19	I get shaky	0.37	0.91
Panic/somatic symptoms				0.89
	27	When I get frightened, I feel like I am choking	0.99	0.86
	6	When I get frightened, I feel like passing out	0.83	0.90
	12	When I get frightened, I feel like I am going crazy.	0.83	0..86
	1	When I feel frightened, it is hard to breathe	0.82	0.86
	38	When I get frightened, I feel dizzy.	0.73	0.86
	34	When I get frightened, I feel like throwing up	0.69	0.86
	24	I get really frightened for no reason at all	0.64	0.87
	30	I am afraid of having anxiety (or panic) attacks	0.62	0.87
	18	When I get frightened, my heart beats fast	0.47	0.87
	22	When I get frightened, I sweat a lot.	0.46	0.87
	15	When I get frightened, I feel like things are not real.	0.42	0.87
Social anxiety				0.88
	32	I feel shy with people I don’t know well	0.93	0.87
	26	It is hard for me to talk with people I don’t know well	0.83	0.87
	41	I am shy	0.80	0.87
	3	I don’t like to be with people I don’t know well	0.69	0.87
	40	I feel nervous about going to parties, dances, or any place where there will be people that I don’t know well	0.67	0.86
	10	I feel nervous with people I don’t know well	0.66	0.86
	39	I feel nervous when I am with other children or adults and I have to do something while they watch me (e.g. read aloud, speak, play a game, play a sport)	0.53	0.88
	4	I get scared if I sleep away from home	0.23	0.89
Separation anxiety				0.78
	25	I am afraid to be alone in the house	0.79	0.73
	29	I don’t like to be away from my family	0.74	0.74
	13	I worry about sleeping alone	0.72	0.73
	16	I have nightmares about something bad happening to my parents	0.67	0.76
	31	I worry that something bad might happen to my parents	0.64	0.74
	8	I follow my mother or father wherever they go	0.53	0.75
School avoidance				0.86
	36	I am scared to go to school	0.90	0.78
	17	I worry about going to school	0.88	0.78
	2	I get headaches when I am at school	0.40	0.83
	11	I get stomachaches at school	0.36	0.87
Total Cronbach’s alpha				0.95

*p* < 0.01

### Factor structure

Factor analysis showed that the Korean version of the SCARED was comprised of five factors, which reflected generalized anxiety (GAD), panic/somatic symptoms (PD), social anxiety (SP), separation anxiety (SAD) and school avoidance (SA) (Table 3). We determined the number of the factors using Cattell’s scree test, which directs the dropping of components after showing a gradient decline in the scree plot. Our scree test suggested retention of five or six factors; thus, analyses were carried out to assess the interpretability and factor structure of both five- and six-factor structures. The six-factor solution resulted in a factor which contained only two variables, which did not satisfy the general principle that a factor is more stable when it contains at least 3 items or more [39]. In addition, the factor did not yield any significant independent factor based on K-SADS-PL nor DSM-V [40]. Therefore, a five-factor structure was chosen for better interpretation [39]. Every item with the exception of item 4, which had a factor loading of 0.23, exceeded the factor loading of 0.30, the minimum required loading of an item [41]. Two items, items 10 and 15, showed cross loading, which means that two or more items had factor loading of more than 0.32 [41], for the GAD factor (0.34 and 0.33, respectively) and the PD factor (0.66 and 0.42, respectively). The explained variance of the factor structure was 58.9%.

The GAD factor consisted of 12 items, and of these items, ‘I worry about people liking me’ had the highest factor loading (1.01). Factor loadings for all items ranged from 0.37 to 1.01. The PD factor consisted of 11 items, and the item ‘When I get frightened, I feel like I am choking’ scored the highest factor loading (0.99). Factor loadings for PD ranged from 0.42 to 0.99. The SP factor was composed of 8 items, and the factor loadings ranged from 0.23 to 0.93. The item ‘I feel shy with people I don’t know well’ showed the highest factor loading. SAD included 6 items and their factor loadings ranged from 0.53 to 0.79. ‘I am afraid to be alone in the house’ showed the greatest factor loading. The SA factor was composed of 4 items with factor loadings of these items ranging from 0.36 to 0.90. (Table 3).

### Comparison of SCARED scores between anxiety group and non-anxiety groups

The comparison of the SCARED scores based on our five-factor structures among the A + D, D only and control groups is presented in Table 4. There were significant group differences in the Korean SCARED scores for generalized anxiety, social anxiety, separation anxiety and school avoidance. The scores of these factors were in the following order: A + D, D only, and control groups. However, A + D and D only group did not show any differences in scores of panic symptoms.

**Table 4:** Comparison of SCARED among Anxiety + Depression(A + D), Depression only(D only), and Control groups

Factor	§ Anxiety + Depression (n = 44)	§§ Depression only (n = 49)	Control (n = 54)	statistics	p-value
Total†	43.57 (15.08)	32.35 (14.85)	10.61 (9.73)	*F* = 79.17	< 0.001
Generalized anxiety†	16.91 (5.12)	13.31 (6.42)	4.26 (4.19)	*F* = 75.77	< 0.001
Panic/somatic symptoms††	6.55 (5.78)	4.47 (4.44)	0.96 (1.45)	*F* = 22.73	< 0.001
Social anxiety†	10.61 (4.04)	7.94 (4.51)	3.83 (3.27)	*F* = 36.93	< 0.001
Separation anxiety†	4.18 (3.30)	2.65 (2.66)	1.06 (1.86)	*F* = 17.31	< 0.001
School avoidance†	5.32 (2.31)	3.63 (2.52)	0.48 (0.97)	*F* = 73.74	< 0.001

§ Anxiety + Depression: participants with co-morbid anxiety disorders and MDD

§§ Depression only: participants with MDD only

† A + D > D only > Control group

†† A + D = D only > Control group

### Sensitivity analysis

The absolute fit of the five-factor SCARED we elicited was tested on the independent clinical sample. The model yielded χ2/df value of 2.63, (χ2 = 2021.13, df = 769, *p*-value < 0.001) which is acceptable [42]. Root Mean Square Error of Approximation (RMSEA) was 0.063 and Standardized Root Mean Square Residual (SRMR) was 0.078, which showed adequate fit to the data [42]. However, relative fit indices showed a moderate fit. The comparative fit index (CFI) was 0.86 and the Tucker-Lewis index (TLI) showed 0.85, which did not reach the optimal cutoff of 0.95 [42, 43]. The standardized factor loadings of the Korean SCARED for the second sample are presented in Table 5. Factor loadings were all higher than 0.36 (Table 5).

**Table 5:** Standardized factor loadings and factor internal consistency on independent dataset (*N* = 431)

Factor	Item		Factor loading	Cronbach’s α
Generalized anxiety				0.91
	5	I worry about other people liking me	0.71	0.90
	21	I worry about things working out for me	0.67	0.90
	33	I worry about what is going to happen in the future	0.72	0.90
	14	I worry about being as good as other kids	0.73	0.90
	23	I am a worrier	0.75	0.90
	28	People tell me that I worry too much	0.69	0.90
	35	I worry about how well I do things	0.74	0.90
	37	I worry about things that have already happened	0.75	0.90
	20	I have nightmares about something bad happening to me	0.60	0.91
	9	People tell me I look nervous	0.58	0.91
	7	I am nervous	0.58	0.91
	19	I get shaky	0.63	0.91
Panic/somatic symptoms				0.89
	27	When I get frightened, I feel like I am choking	0.81	0.86
	6	When I get frightened, I feel like passing out	0.36	0.90
	12	When I get frightened, I feel like I am going crazy.	0.77	0.86
	1	When I feel frightened, it is hard to breathe	0.75	0.86
	38	When I get frightened, I feel dizzy.	0.76	0.86
	34	When I get frightened, I feel like throwing up	0.71	0.86
	24	I get really frightened for no reason at all	0.69	0.86
	30	I am afraid of having anxiety (or panic) attacks	0.62	0.87
	18	When I get frightened, my heart beats fast	0.64	0.87
	22	When I get frightened, I sweat a lot.	0.55	0.87
	15	When I get frightened, I feel like things are not real.	0.63	0.87
Social anxiety				0.88
	32	I feel shy with people I don’t know well	0.73	0.86
	26	It is hard for me to talk with people I don’t know well	0.71	0.86
	41	I am shy	0.73	0.86
	3	I don’t like to be with people I don’t know well	0.69	0.87
	40	I feel nervous about going to parties, dances, or any place where there will be people that I don’t know well	0.80	0.86
	10	I feel nervous with people I don’t know well	0.81	0.86
	39	I feel nervous when I am with other children or adults and I have to do something while they watch me (e.g. read aloud, speak, play a game, play a sport)	0.66	0.87
	4	I get scared if I sleep away from home	0.49	0.89
Separation anxiety				0.76
	25	I am afraid to be alone in the house	0.72	0.73
	29	I don’t like to be away from my family	0.57	0.74
	13	I worry about sleeping alone	0.66	0.73
	16	I have nightmares about something bad happening to my parents	0.54	0.76
	31	I worry that something bad might happen to my parents	0.58	0.74
	8	I follow my mother or father wherever they go	0.54	0.75
School avoidance				0.86
	36	I am scared to go to school	0.93	0.78
	17	I worry about going to school	0.93	0.78
	2	I get headaches when I am at school	0.65	0.83
	11	I get stomachaches at school	0.56	0.87
Total Cronbach’s alpha				0.95

*p* < 0.01

## Discussion

As expected, our results showed that the Korean SCARED showed high internal consistency with each subscale demonstrating high internal consistency. This finding is consistent with other validation studies of the English SCARED done in clinical samples, in which Cronbach’s α of subscales ranged from 0.79 to 0.89 [6].

The Korean SCARED also demonstrated good criterion validity. The Korean SCARED total scores had a high correlation with K-SADS-PL anxiety subscales, and a moderate correlation with the CBCL anxious/depressed subscale and the CBCL internalizing scale. These findings may imply the discrepancies between the reports of the participants and their parents because the CBCL was completed by parents. Parents tend to under-report their children’s internalizing symptoms such as depression and anxiety compared to externalizing symptoms because the latter are more evident to parents [44]. In addition, the CBCL anxious/depressed subscale not only measures anxiety, but also depressive symptoms, which may have contributed to the weakened correlation between the SCARED and the CBCL anxious/depressed subscale. Moreover, the Korean SCARED was not associated with the CBCL externalizing scale, CD of the DBD or ADHD-RS, which supports high discriminant validity of the Korean SCARED. Taken together, the Korean SCARED would be a reliable and valid screening tool for anxiety symptoms in youth.

In our sample, exploratory factor analysis produced five factors: GAD, PD, SP, SAD, and SA. This factor structure was comparable to the factor structures produced in other cultures, which generally showed four (GAD, PD, SP, and SAD) to five (GAD, PD, SP, SAD, and SA) factors [5, 6, 18, 45–47]. The explained variance in our factor structure (58.9%) was higher than the explained variances from other five-factor solution studies, which ranged between 36.3 and 39.5% [18]. The result might reflect that the factor structure of the Korean SCARED explains anxiety disorders in Korea youth well. However, the prominent difference in the explained variance value can be due to our sample characteristics, which was a clinical sample with a high proportion of the youth having mood problems, whereas the majority of previous SCARED factor analyses were conducted with a community sample. There were differences in some items that comprise the factors compared to Birmaher’s factor structure [5]. Item 4, ‘I get scared when I sleep away from home’, belonged to social anxiety rather than separation anxiety as identified in Birmaher’s study [5]. Item 9, ‘People tell me I look nervous’, and 19, ‘I get shaky’ were included in the generalized anxiety factor, not panic disorders as in Birmaher’s analysis. Finally, item 20, ‘I have nightmares about something bad happening to me’ belonged to generalized anxiety, not separation anxiety as in the original study.

As for item 4, the difference in factor structure may be due to the different age range of participants. In contrast to Birmaher’s study which included children from the age of 9 to 18 years old, our participants were all adolescents, with the age range between 12 to 17 years [5]. Rates of separation anxiety are highest in younger children [48]. However, the SCARED factor structures found in the research of different countries with similar age ranges as our study, resemble the original study by Birmaher. In those studies, item 4 was included in SAD [45, 49]. Another reason for the different results may be related to the cultural differences between South Korea and the United States. For example, in South Korea, sleeping away from home could be because of school retreats (Suhak Yeohaeng in Korean), an educational activity that students are required to attend once or twice a year. This event is conceptually different from the western sports camps in that it requires obligatory participation. At the school retreat, Korean children are often required to collaborate with unfamiliar classmates. In this context, it is possible that a Korean child may interpret item 4 as a situation in which unwanted social interactions are required. This may cause them to have worries about how other people view them, ‘Nun-chi’ in Korean, and social fear, ‘Taein Kongpo’ in Korean. Consistent with this interpretation, item 4 was included in the social phobia factor, highly correlating with items such as ‘I am shy’, ‘I feel shy with people I don’t know well’ and ‘I feel nervous about going to parties, dances, or any place where there will be people that I don’t know well’. In addition, factor loading was relatively low (0.23) compared to other items, which might partially explain the different result.

Another example showing the cultural difference is that item 9, ‘people tell me I look nervous’, can be thought of as a question of how adolescents perceive others’ view of them. GAD worries tend to strongly focus on interpersonal difficulties [50] and prominent self-consciousness [51]. The fact that item 4 and 9 belonged to a different factor compared to Birmaher’s original study [5] may reflect the Korean cultural tendency to worry about other people’s views and assessments rather than their own sense of the self, panic or somatic symptoms. Therefore, these differences in the items comprising the factors might reflect the cultural differences between South Korea and the U.S.

The social anxiety scores prominently differed between our sample and the US clinical sample. The mean scores of A + D group (10.6) and D only group (7.9) in our sample were higher than those from a study with a corresponding US clinical sample (3.7 in anxiety only group and 2.7 in depression only group) [5]. This indicates that Korean adolescents tend to feel more anxious in interpersonal and social situations. This result is in line with the above-mentioned result that Korean adolescents tend to understand item 4 as a social interaction related anxiety rather than separation-related anxiety.

We further investigated how the Korean SCARED can be used to discriminate the A + D, D only and control groups, according to the elicited factor structures. All of the anxiety subscales, excluding PD, significantly distinguished the three groups: scores were the highest in the A + D group and the lowest in the control group. The PD subscale score also followed the same order, but the difference between the A + D and the D only group was not significant. Although the panic disorder is categorized as an anxiety disorder according to DSM-5 [30], depressive disorder is also highly comorbid with panic symptoms [52]. In this sense, the Korean SCARED could not clearly distinguish the A + D comorbid group from the D only group in terms of PD. Nevertheless, the PD subscale distinguished the A + D and the D only groups from the control group. In this respect, the Korean SCARED might be useful as an anxiety assessing tool that can fairly discriminate between the subcategories of anxiety disorders.

Sensitivity analyses demonstrated that our factor structure had a good fit measured by absolute fit indices, SRMR and RMSEA, but a moderate fit by relative fit indices, CFI and TLI. Several factors may have influenced the inadequacy of relative fit indices. First, limitations of our factor structure may have influenced the results, because there were items with cross-loadings (e.g. item 10 and 15) and low factor loading (e.g. item 4). Second, sample size of our primary sample may have affected the results, which met the minimal sample size needed for the factor analysis, but not the optimal size [38]. In addition, composition difference between our primary sample and the independent clinical sample may have affected the results. Our sample was composed of youths with mood disorders and healthy controls, whereas all but one of the independent clinical sample had psychiatric disorders. Our results of low CFI/TLI values and adequate SRMR/RMSEA of our factor structure may be due to the fact that the CFI/TLI are relative fit indices, while the SRMR/RMSEA are absolute indices. Relative fit indices compare the model with null hypothesis, which assumes that all variables are not correlated [53]. Lower than optimal value of relative fit indices may reflect the moderate correlations between inter-factor items, although intra-factor items were highly correlated [54]. However, absolute fit indices are determined by how far the model is from the perfect fit, which is presumed to have a fit of zero [42, 53, 55]. The adequate SRMR suggested that our factor structure captures the data well, and the good RMSEA index indicated that our model fits well relative to degree of freedom [54], which indicates that our factor structure is adaptable to clinical samples. Different modeling formulas seem to have caused the difference in the results, lowering the relative fit indices.

There are several limitations to this study. The sample of the study was not randomly selected from the general population, and depressed patients were recruited from an outpatient clinic. This may obscure the generalizability of the results to the general population. However, we included a control group that can mitigate selection bias. Additionally, participants’ ages were between 12 to 17 years, which can limit the generalizability to younger children. However, sensitivity analysis suggests that the factor structure can be applied to a wider age range of children and adolescents. Another limitation is that parents completed all the questionnaires with the exception of the Korean SCARED, and their reports may significantly differ from the participants’ report regarding the internalizing symptoms such as anxiety and depression [44]. Nevertheless, there are some studies about child and parent agreement that revealed concordance between child and parent reports to be moderate to high [56, 57]. This study was also limited in that the anxiety only group was not included and only the anxious/depressed comorbid group was included in the analysis. Despite this limitation, it is important to note that anxiety disorders are highly comorbid with other psychiatric disorders in adolescents, and thus discriminating anxiety only subjects may be a challenging task. Lastly, the small sample size was another limitation of our study. However, our sample size met the minimum sample size for factor analysis as suggested by Mundfrom [38] and other studies, which state that sample size should be at least 3 times the number of variables or a minimum size of 100 [58, 59].

Despite the above-noted limitations, our study showed that the Korean SCARED is a reliable and valid screening tool for assessing adolescent anxiety with high criterion and discriminant validity. Taking into account the high prevalence and the negative influence on the daily function of adolescents’ anxiety disorders, the Korean SCARED can serve as a utilitarian method to screen anxiety disorders and provide a diagnostic impression about the types of anxiety disorders. Furthermore, investigation of the factor structure suggested [5] a cultural difference of anxiety symptoms between Korean and Western adolescents. Future studies that utilize the SCARED to explore sub-anxiety differences among countries are needed, which might reveal additional cultural differences.

## Conclusion

Our study suggests that the Korean SCARED is a promising screening tool for assessing adolescent anxiety to be used practically in community settings as well as clinical environments including primary care. The fact that different items comprised the factors may reflect cultural differences in experiencing anxiety between youth in the United States and Korea; Korean youth report higher social fears and worry about how other people view them.

## Data Availability

The datasets used and/or analysed during the current study are available from the corresponding author on reasonable request.

## Abbreviations

A + D (Anxiety + Depression): Participants with co-morbid anxiety disorders and MDD
ADHD-RS: ADHD Rating Scale
CBCL: Child Behavior Checklist
CDRS-R: Children’s Depression Rating Scale-Revised
CFI: Comparative fit index
D only: Participants with MDD only
DBD: Disruptive Behavioral Disorder Scale
DSM-5: Diagnostic and Statistical Manual of Mental Disorders-5th Edition
GAD: Generalized anxiety
IQ: Intelligence quotient
K-SADS-PL: Kiddie-Schedule for Affective Disorders and Schizophrenia for School-Age Children-Present and Lifetime Version
MDD: Major depressive disorder
PD: Panic/somatic symptoms
RCMAS: Revised Children’s Manifest Anxiety Scale (RCMAS)
RMSEA: Root Mean Square Error of Approximation
SA: School avoidance
SAD: Separation anxiety
SCARED: Screen for Child Anxiety Related Emotional Disorders
SP: Social anxiety
SRMR: Standardized Root Mean Square Residual
STAIC: State-Trait Anxiety Inventory
TLI: Tucker-Lewis index
